# American Academy of Dental Science

**Published:** 1900-03

**Authors:** 

**Affiliations:** American Academy of Dental Science


### AMERICAN ACADEMY OF DENTAL SCIENCE.

THE regular monthly meeting of the American Academy of Dental Science was held at Young's Hotel, Wednesday evening, October 4, 1899, at 6 o'clock.

A paper was read by C. N. Johnson, L.D.S., D.D.S., of Chicago, Ill., entitled "Filling-materials."

(For Dr. Johnson's paper, see page 145.)

### DISCUSSION ON DR. JOHNSON'S PAPER.

*President Cooke.*—Several years ago, one of our members enjoyed a very pleasant time with Dr. Johnson in his office. He told me this evening that was one of the pleasantest recollections he had of his trip to Chicago. I will call upon Dr. Andrews to open the discussion of this subject.

*Dr. Andrews.*—I remember with pleasure, and I have recalled many times, the pleasant hours I spent with Dr. Johnson in his office, many years ago, in Chicago, and it is a delightful occasion to me to meet Dr. Johnson and to hear his able paper.

I do not know that there is a great deal to be said on the subject that he has not covered. One point which pleased me was his reference to the use of platinum and gold-foil for filling the anterior teeth. I have used it for a number of years, and I have had much satisfaction resulting from the success of these fillings. You can nearly approximate the color of the teeth with one of the three preparations that are furnished us by the dental dealers, and I can fully indorse all that the essayist has said in favor of these materials.

I wish Dr. Johnson could tell us something about successful cements. I think cement is one of the most difficult things we have to deal with,—finding the proper kind. I have tried all kinds without getting anything that could be relied on. I have been for a great many years an advocate of preparatory fillings in young, frail teeth,—filling such teeth with cement. The teeth seem to be better prepared by such treatment for permanent work. I believe in this preparatory work to-day. I have seen the value of it after an experience of over thirty years. I advocated this treatment before the Massachusetts Dental Society many years ago, and the late Professor Chandler sat down very severely on me. But I had the satisfaction of hearing him some dozen years afterwards advocating this same method. We are on the watch for the coming cement. Every kind that I have tried has its faults. In some cases the cement seemed to do wonderfully good service, almost permanent work. I have known some to last for ten years, and that is exceedingly satisfactory, but the same cement in another mouth may fail in six months. You cannot depend on any cement that I know of. And this is the trouble in regard to inlays, you are never quite sure how long they will remain in spite of the utmost care in fitting your inlay. I have had inlays that did good service for several years; others, which have been put in with care, have loosened within six months. The inlays make very beautiful fillings, but the great trouble is, you cannot depend upon your cement. I can indorse anything he has said about gold.

I think amalgam has a larger claim on our good opinion than Dr. Johnson concedes to it. I regard Dr. Johnson as an excellent authority, but I do believe in a good amalgam filling in the place where it is indicated. I have seen in my own practice amalgam fillings that have stood the test of twenty and thirty years in the distal portions of the mouth, and I think these have done quite as good service as gold. They do not look as handsome, perhaps, but in places where they are not seen they are very satisfactory. We must use judgment in what we use and how we use it.

*Dr. Fillebrown.*—I have had the privilege for a number of years of instructing young men, and I have invariably taken the ground that gold was the best all-round filling that we had, and, barring its color, suitable in every way. The matter of conductivity does not count very much against it, because teeth that are normally constructed and not decayed near the pulp will bear all the thermal changes that will come through any conductivity of the gold, and if the cavity is large enough to imperil the pulp, the difficulty can be overcome by laying over it some non-conductor, but not cement, because it is very likely to kill the pulp, no matter what you use for the rest of the filling.

We have one man among us in the city that I remember as long ago as 1869, who took the ground that it was not thermal changes that killed the pulp, and gold was the best metal to come in contact with it. I refer to Dr. N. W. Hawes, and he at that time was placing a few thicknesses of gold-foil in the bottom of the cavity and gently burnishing it down, being very careful to exclude the air and not to put any pressure on the pulp. He claimed that he got the best results in that way,—better than trying to put any other substance over it. I have not thought to ask him what his later experience has been.

I do not think the exposure of gold is particularly objectionable, although I have long recognized the fact, without trying to find a reason for it, that in some mouths it looked well, while in others it was not quite satisfactory, and might be said to appear obtrusive. I think the essayist has given the analysis of that difference to-night. At the same time gold can be used in many cavities in the anterior teeth without any objectionable exposure of the gold. All of us who follow the lines laid down by Dr. Arthur a good many years ago,—trimming the lingual surfaces of the cavity to the lingual aspect instead of to the labial aspect, will put in a filling that will be scarcely noticeable. So long as operators will insist on inserting fillings from the labial aspect, just so long they will make an objectionable exposure of gold. I have always tried to encourage the young men,—and the older ones, too,—by that means to keep the amount of gold that will show down to the smallest possible point. While Dr. Arthur's methods have been criticised a great deal, in my hands they have proved exceedingly satisfactory, and I have noticed it in our classes, that those students who thoroughly comprehend that idea and most carefully carry it out, are the ones who get the best results æsthetically and physiologically.

I favor the use of platinum and gold. I have a patient for whom I put in a platinum and gold filling in 1875 and 1876, and it is in excellent condition to-day. The objection I had to that was the color, the gold having little effect in lightening up the platinum. Being so favorably impressed with it, I entered into correspondence with manufacturers, telling them what I wanted, and I got one or two samples in answer to this correspondence, which were better than what I had used, but not as satisfactory as I had hoped for, consequently I did not give it the use that I might have otherwise. I was aiming for just the very thing Professor Johnson speaks of,—taking off a little of the gold tint without losing all of its color. I am glad to know that it is made practicable and can now be done.

Some of the remarks of the essayist seemed to be based upon the idea that all gold must be malleted. It was not a positive statement, but by inference from his remark that the reason for not using gold in certain places was that it would not bear malleting. Now, perhaps the gentleman would be surprised if he should go down to the Harvard School, where we have seventy or eighty men at work, and hardly a mallet in use there. We expect every man to have a mallet and to know how to use it, but it is not in one filling out of ten that the mallet is used in making gold fillings, and I venture to say that the fillings will pass muster in most cases. A number of years ago I used to think that if a gold filling was not malleted, it was not good for anything. I have changed my opinion very largely in respect to that, and I now believe that if a man has a set of the new Harvard pluggers (which were gotten up within the last year by the combined wisdom of all the Harvard instructors) and can use them with a tolerable degree of skill, he will be convinced that the mallet's mission has been largely curtailed.

*Dr. Eames.*—I would like to ask Dr. Fillebrown, when he speaks of not using the mallet, just what he does do? Is it a rubbing motion with the hand pressure, or just a direct push?

*Dr. Fillebrown.*—In most cases it is hand pressure with serrated points. We do use the smooth points and burnish on, especially in finishing. A series of clinics was given some time ago at which the filling was done altogether by burnishing. It was called the

Shumway-Libby Improved Method. The most of the work is hand pressure, and working the gold cohesively.

*Dr. Hamilton.*—I like to say a good word for tin and gold. My contour work always commences with tin and gold at the cervical wall. It has been satisfactory there and I was pleased to hear the essayist give it his commendation. I wish every dentist would try it and be convinced of its great value.

*President Cooke.*—How much do you use of the tin and gold in such cases?

*Dr. Hamilton.*—I use about one-third up the contour where it will not show. If it is in the bicuspid where a side view. comes, I place in one fair layer of the combination of one-half tin and one-half gold.

*Dr. Gillett.*—In general, the principles laid down by the essayist meet my views so thoroughly that I have little to add. There are three points which I wish to mention.

I wish to express my particular satisfaction with what he had to say in regard to the removal of soft decay from the bottom of cavities. There is such a wide-spread belief in the practicability of leaving decayed dentine at the bottom of the cavity, that to oppose this practice is regarded in many quarters as rank heresy, but I have seen such ill results following it that I believe it a point to be emphasized, that we should be satisfied only with thorough excavation of decayed dentine. I have no sympathy whatever with the statement that it is better to leave it than to expose the pulp. A large majority of such pulps give serious trouble afterward. They are apt to be constantly irritable, even if they do not die in the end. It so often results in having to undo what would otherwise be satisfactory work, and in serious suffering and injury for the patient, that it seems to me we ought to make ourselves plain on that point. I was glad to hear Dr. Johnson take the stand which he did.

One combination which we apparently think better of than they do in Chicago, is that of amalgam and gold. I recognize that there are certain objections to its use. On the other hand, I have had such satisfactory results, in selected cases, that I cannot but think well of it, and as the quality of our amalgam grows better,—and I think it has grown better in the last few years,—my use of that combination has been rather extending than otherwise. I have such fillings on record in satisfactory condition after ten years of service.

What he has said about the harmonizing of the gold and platinum in the features of the brunette, brings forcibly before us one of the valuable points in the use of that combination. It explains to me why I have been particularly well pleased with a certain filling in the mouth of a decided brunette. It has been a constant surprise to me that it looked so well, and this point may be borne in mind with benefit in our future operations.

There is one point which I wish Dr. Fillebrown would make plain or amplify a bit. If I understood him, he said that cement always kills the pulp,—is that your belief?

*Dr. Fillebrown.*—Almost always. It generally injures it.

*Dr. Gillett.*—Do you mean in the cases of exposed pulp, or moderately large cavities which come near being exposed,—where do you draw the line?

*Dr. Fillebrown.*—I think in my case, where the cement is placed very near to the pulp, the pulp is likely to be affected by it.

*Dr. Gillett.*—Suppose there was a fair layer of sound dentine between it and the pulp?

*Dr. Fillebrown.*—The pulp would probably stand it in that case.

*Dr. Gillett.*—That point has been much discussed and in some places the consensus of opinion seems to coincide with that of Dr. Fillebrown. I believe that most exposed pulps die under any filling, and I include in this all those where the decay has actually perforated the pulp chamber, whether the decayed dentine is removed or left by the operator. I am equally pronounced in my belief that the zinc phosphate cements have no ill effects in cavities where the pulp has a reasonably thick covering of sound dentine. During all my professional life it has been my habit to place zinc phosphate under all gold and amalgam fillings when the cavity was deep enough to permit of so doing, and I have yet to meet the case in which I thought the pulp was disturbed by the action of the cement *per se*. It is a matter regarding which there are widely divergent views in the profession, and one which may well have investigation to determine which opinion is right. If either view is the correct one, it is important that its correctness be established.

*Dr. Hopkins.*—There was one point which the essayist brought out very strongly, and which was referred to later, namely: The matter of leaving decayed dentine under a filling. For my own interest and satisfaction, I filled a number of recently extracted teeth some time ago, in which the nerve was supposed to be nearly exposed, leaving a small part of the decayed dentine at the bottom of the cavity. The cavity was washed out with carbolic acid and absolute alcohol, and sterilized as completely as any sterilization could be carried in the mouth. I then filled the cavities, and in three weeks removed the fillings and the dentine at the bottom of the cavities and put them in culture tubes to see whether there were any bacteria still living and thriving. In every instance I had cultures, showing that the sterilization such as I had given, and which was as thorough as it could possibly be done in the mouth, had not destroyed the bacteria in that decayed dentine at the bottom of the cavity.

*Dr. Andrews.*—Were the dead pulps allowed to remain in the teeth?

*Dr. Hopkins.*—No, the roots of the teeth were sawed off, the pulps removed, and the pulp canals cleansed; the teeth were filled on the root side as well, but the wall between the pulp cavity and the fillings still remained intact,—no absolute exposure.

*Dr. Fillebrown.*—I would like to say a word explanatory of my statement regarding the action of cements on the teeth. I can only say that my observations, which began in 1869 and have been continuous to 1899, have led me to the conclusion which I have stated here to-night,—that the action of cement in the teeth is injurious, and if it be so near the pulp that it cannot resist those injurious effects, you are liable to have trouble. I once advocated, and should not oppose now, leaving decalcified dentine over the pulp. I have always removed all decayed dentine, but simply decalcified dentine I thought it safe to leave. I have always advocated the removal of all decayed dentine for reasons so succinctly stated by Dr. Gillett, and believe that if the pulp is in danger of exposure by the removal of decayed or disorganized dentine, then the pulp had better be destroyed at once, for it is bound to die anyhow. I am satisfied that a great many pulps die which do not come to our notice, and because the patient does not come to us, we believe that our cement fillings have been successful.

*Dr. Andrews.*—They die under gold or amalgam at times.

*Dr. Fillebrown*—Certainly, if you approach near enough to it the pulp is likely to be affected by the presence of any filling, cement, or anything else, and is pretty sure to die; and for that reason I believe that if a tooth is so far decayed that the removal of the decayed and disorganized dentine is going to expose the pulp, it had better be destroyed at once.

*Dr. Williams.*—One mistake, I think, is generally made in regard to the treatment of decayed teeth with cements, and the disappointments resulting from it are in overlooking the fact that the cement becomes hard, flinty, in the interior of the tooth, where the tissues are all more or less elastic, and the minute pulsations of the pulp rebound against it, producing an irritation. We should first use some antacid like lime-water for the correction of the acid conditions of the cavity, and an antiseptic like creosote used to be one of the most reliable; and we now have a good form of it in guaiacol, which is not so strong. We can then proceed with the stopping, taking care to have the material correspond more in density with the density of that portion of the tooth, and the pulsations of the pulp will not be so firmly resisted and cause so much irritation as by the method which is most commonly employed at present, which is to first place in a piece of thin paper and then put the cement on that. The phosphate becomes hard and rocky, and I attribute the death of a great many pulps to that cause. Instead of having some plastic material, which would correspond more with the density of that part of the tooth, I have seen persons who thought it was all right to hammer gold fillings in as hard as possible, and under such treatment it is not remarkable that pulps not nearly exposed would die.

Years ago, when I was a youngster, I devised a treatment for such cases which I called a "preparatory treatment." At that time many were using gutta-percha or Hill's stopping (which was a mixture of gutta-percha and plaster-of-Paris). The idea occurred to me that the oxide of zinc might be used, so I used that as a basis for the plastic. Even then the gutta-percha would have a certain elasticity and expand so as to press against the pulp. A mixture of gutta-percha filling and beeswax made a very yielding material to put into the bottom of the cavity, and I have succeeded in saving a great many teeth that seemed to be very near pulp exposure. One particular case that I remember was a cavity in the mesial surface of a left, upper first molar. I gave it the preservative treatment which I was using at that time, which was to first saturate the cavity with chloride of lime solution (I also used simple *aqua calcis* in some sensitive cases), then perhaps saturate with a slight solution of tannin mixed with a little dilute creosote, some soft material that acted as a sort of a sponge for the antisepsis of the pulp, and over it was placed the mixture of beeswax and gutta-percha. After it had remained there for six months without giving any trouble whatever, I removed the stopping and soft material until I came to a substance at the bottom of the cavity that looked like dentine, and as I passed my probe over it I found it hard. Ossification had taken place under that very soft material. A year afterward the same tooth decayed on the distal surface, and when I came to fill the cavity, I found the dentine quite sensitive, showing that the former treatment had been successful. I went on with that treatment at a time when many were filling with gold. One day one of my patients happened to call with a friend on a certain dentist, famous for gold filling, and mentioning my treatment he asked to see one of my preparatory fillings, and said he was surprised that Dr. Williams would put such stuff as that in teeth, and that nothing but gold should be used. In those days dentists did not meet and discuss matters as we do now, and, in fact, they tried to keep secret whatever successful methods they used themselves. Eventually I had occasion to meet the old gentleman, who at that time was using nothing but gold, and in the course of our conversation, I told him that I thought that practice was in the same line with the man who pretended to practice medicine and had only one medicine to cure all diseases. He said that he would not fill a tooth that he could not fill with gold. He selected only those that promised to hold the gold fillings, the others he would take out. I told him that was like the physician who had a reputation of curing all his patients, but the secret of his success was the fact that he selected only those patients who would get well. Finally, in the course of time, I got him to acknowledge that that class of fillings had its uses, and that they were sometimes useful.

I think Dr. Johnson in his very exhaustive paper has given a very good sketch of the various materials in their usefulness. We are handicapped by the lack of a durable cement by which porcelain fillings can be retained permanently. They now seem the most ideal filling for a conspicuous cavity, the inlay style, because there is nothing which more nearly resembles the tooth, and I could never find, with all the metals, gold, and the various combinations with gold, anything which would harmonize in appearance with the tooth so well as the inlay. It was claimed that some of the whitened amalgams would do it, but I do not know how long they remained white.

However successful we may be with any material, we must remember that no one thing is a "cure-all," no one thing is the best in all cases, as Dr. Johnson has suggested. We must try and select what is best adapted for the particular case in hand, physically as well as surgically and mechanically.

*Dr. Andrews.*—I remember very well the condition of affairs which Dr. Williams has spoken of in older days. The laboratory was then the dentist's sanctum sanctorum, and one dentist would not think of allowing another into his laboratory. He might get at some of his secrets or find out his methods.

In regard to the treatment of exposed pulps, you must use the judgment I spoke of a little while ago. You must select your patient and select young ones. I have had success with adult patients, but it is seldom wise to attempt it, unless there is a great deal of vigor and health apparent in the patient. One of my earliest cases of treating an exposed pulp was for a student studying with me, and in that case the pulp was clearly exposed, so that it could be seen pulsating. I made an application of lactophosphate of lime, and then inserted an oxychloride filling over that, and two years afterward the filling was all removed, the pulp was covered with secondary dentine and a gold filling was malleted into that cavity by Dr. Weatherbee, at a clinic in Tremont Temple, at a meeting of the Massachusetts Dental Society. That was years ago, and the tooth is alive to-day.

I wish to speak of the use of the Scotch stone in finishing platinum and gold-foil fillings. If you will use it in the final finish of your gold and platinum-foil fillings, you will be surprised to see how near those fillings will approximate the "pearl" that Dr. Johnson has spoken of.

*Dr. Johnson.*—I do not know that I should take up any more of your time. I was aware that my paper was a long one, and I hurried through with it on that account, but some points have come up in the discussion to which I should like to refer.

Dr. Andrews's plea for a cement which is reliable is something which strikes very near to all of us. I have been earnestly hoping for years that we should finally discover such a material, but I also regret to say that in the light of some recent investigations which have been carried on, relative to the physical properties of cements, I do not believe it possible, with our present knowledge, and in view of the conditions of the mouth, to secure in the immediate future a material of that nature which we can classify as a permanent filling material. This does not imply that no improvement is being made in our cements; on the contrary, there is a hopeful advance by some of our manufacturers, who are studying the matter scientifically, and we have great promise of a much improved product.

As to the distinction between hand pressure and the mallet in the insertion of gold, I consider it an important subject. Early in my practice I became afraid of the mallet on account of injuries that I saw worked by its use, so for years I used nothing but hand pressure, and I think I accomplished fairly good results. But we must not lose sight of the demonstrated fact, that with a given amount of force, gold may be made denser and harder with the mallet than with the hand, and to-day I feel that I need mallet-force, particularly upon the surfaces of my fillings.

As to the combination of amalgam and gold mentioned by Dr. Gillett, I have seen beautiful results where it has been employed, filling the cervical third with amalgam and finishing with gold, but I have not practised that method myself, because I cannot work amalgam, in connection with gold, to my satisfaction. I cannot mallet gold upon amalgam with the same assurance that I can upon tin and gold. It is a matter of personal preference with me. I can take gold and tin, rolled together, and build the cervical third of a filling in one-half the time that I can with amalgam, and then I can go right on with the rest of the operation at the same sitting, and not be hampered by any doubts as to good union between the combination and the gold which composes the larger part of the filling.

There is one thing that I should like to ask Dr. Fillebrown, and that is, on what basis he is inclined to claim that cements necessarily injure the tooth when in close proximity, I do not mean in actual contact to the pulp? I came down here to Boston to learn something, and mean to carry away all I can. I have heard more or less discussion as to the injurious effects of cements upon the teeth, and I should like to ask the gentleman to what cause he ascribes these effects, is it by reason of some irritant in the fluid of the cement?

*Dr. Fillebrown.*—I should say that it must be something of that nature. I have observed this fact for many years, and my theory in regard to it is that there is something in the make-up of the cement that is injurious to the pulp.

*Dr. Johnson.*—I did not know but he might have held the opinion that the arsenic in the cement was working the destruction of the pulp. I have heard the opinion expressed that it was not a safe procedure to put cement in close proximity to the pulp on account of the action of the arsenic contained in the fluid. Now, some tests have been made in Chicago by Dr. Ames which go conclusively to prove that there is not enough arsenic, or rather that it is not in such a form, as to cause irritation to the pulp sufficient to bring about its destruction. There are many pulps that will not tolerate cement in their immediate vicinity, even if there is not a direct exposure, while there are others that will not only submit to it, but are actually benefited by it, being thereby stimulated to the production of secondary dentine, and consequently better protected. I believe that question is largely one of locality, which has been referred to this evening by Dr. Gillett. I believe that there are certain localities in which a pulp that is nearly exposed will not live under any filling or treatment that we at present have knowledge of, and I think this question of locality ought to be taken into consideration. It explains to us why it is that men differ so materially in their reports and opinions concerning such cases.

I trust you will pardon me if, in conclusion, I say a few things to you outside of professional topics. This is my first visit to Boston, and I wish to say that I conceive it to be a very great honor to be invited here to-night; and I also wish to thank you for the kind things you have said and for the welcome you have given me.

I have always wished to visit Boston because of the historic interest connected with it. We have some things of interest in Chicago, but we lack an extended history, and I for one miss that very much. I like to look upon the landmarks that go to punctuate the history of our country. I never think of Boston without thinking of Bunker Hill, and the names of the great teachers, Longfellow, Emerson, and Oliver Wendell Holmes. If all is well, this is not my last visit to Boston, and I wish to extend an invitation to you to come to Chicago. We have not the old associations that you have here, but we shall be glad to see you and make you welcome.

*Dr. Eames.*—I feel personally indebted to Dr. Johnson, our essayist, for coming here to speak to us, and I know that we shall all be glad to have him call upon us in our offices. I take pleasure in moving that we extend our thanks for his most excellent paper, and his visit to us.

Unanimously voted.

Harry E. Cutter, D.D.S.,
*Editor American Academy of Dental Science.*

